# The mental health and wellbeing of first generation migrants: a systematic-narrative review of reviews

**DOI:** 10.1186/s12992-016-0187-3

**Published:** 2016-08-25

**Authors:** Ciara Close, Anne Kouvonen, Tania Bosqui, Kishan Patel, Dermot O’Reilly, Michael Donnelly

**Affiliations:** 1 Administrative Data Research Centre – Northern Ireland, Centre for Public Health, Queen’s University Belfast, Belfast, UK; 2 UKCRC Centre of Excellence for Public Health, Queen’s University Belfast, Belfast, UK; 3 Department of Social Research, University of Helsinki, Helsinki, Finland; 4 SWPS University of Social Sciences and Humanities, Faculty in Wroclaw, Wroclaw, Poland; 5 College of Liberal Arts and Social Sciences, University of Guam, Mangilao, Guam

**Keywords:** Mental health, First generation migrants, Prevalence, Risk factors

## Abstract

**Background:**

First generation migrants are reportedly at higher risk of mental ill-health compared to the settled population. This paper systematically reviews and synthesizes all reviews on the mental health of first generation migrants in order to appraise the risk factors for, and explain differences in, the mental health of this population.

**Methods:**

Scientific databases were searched for systematic reviews (inception-November 2015) which provided quantitative data on the mental ill-health of first generation migrants and associated risk factors. Two reviewers screened titles, abstracts and full text papers for their suitability against pre-specified criteria, methodological quality was assessed.

**Results:**

One thousand eight hundred twenty articles were identified, eight met inclusion criteria, which were all moderate or low quality. Depression was mostly higher in first generation migrants in general, and in refugees/asylum seekers when analysed separately. However, for both groups there was wide variation in prevalence rates, from 5 to 44 % compared with prevalence rates of 8–12 % in the general population. Post-Traumatic Stress Disorder prevalence was higher for both first generation migrants in general and for refugees/asylum seekers compared with the settled majority. Post-Traumatic Stress Disorder prevalence in first generation migrants in general and refugees/ asylum seekers ranged from 9 to 36 % compared with reported prevalence rates of 1–2 % in the general population. Few studies presented anxiety prevalence rates in first generation migrants and there was wide variation in those that did. Prevalence ranged from 4 to 40 % compared with reported prevalence of 5 % in the general population. Two reviews assessed the psychotic disorder risk, reporting this was two to three times more likely in adult first generation migrants. However, one review on the risk of schizophrenia in refugees reported similar prevalence rates (2 %) to estimates of prevalence among the settled majority (3 %). Risk factors for mental ill-health included low Gross National Product in the host country, downward social mobility, country of origin, and host country.

**Conclusion:**

First generation migrants may be at increased risk of mental illness and public health policy must account for this and influencing factors. High quality research in the area is urgently needed as is the use of culturally specific validated measurement tools for assessing migrant mental health.

**Electronic supplementary material:**

The online version of this article (doi:10.1186/s12992-016-0187-3) contains supplementary material, which is available to authorized users.

## Background

Migration occurs across the globe due to a range of factors such as conflict, unemployment and poverty. Although migration is not a recent phenomenon and has been an important part of humanities history [1], research into its impact on health has only gained momentum in the last few decades whilst research into the mental health of migrants remains relatively sparse.

The limited available research has shown a higher prevalence of common mental disorders (CMDs) and higher psychiatric admission rates in migrant communities compared to the majority settled population [2–4]. Initially, there appears to be the ‘healthy migrant effect’, where migrants tend to be among the healthiest of their original population [5]. However, this effect seems to disappear soon after migration and poorer mental health is observed in subsequent generations [4, 6]. Recently, research has investigated migration and mental ill-health and multiple factors including political, socio-economic, geographical, psychosocial, demographic and occupational factors.

Estimating the risk of mental ill-health among migrants and gaining an understanding of the factors which impact upon migrant mental health will facilitate the identification of vulnerable individuals and groups and, in turn, enable improved public health responses to suit the needs of the population. Reviews of investigations of the mental health of migrants have been conducted in different countries and adjusted for different factors [7–11]. This review of reviews evaluates and summarises the current evidence from systematic reviews on the prevalence and risk of mental ill-health in first generation migrants as well as potential explanatory factors.

## Methods

There is no universally agreed definition of the term ‘migrant’. The United Nations definition of a migrant without the restriction on length of residence was used in this review, as, the definition of migrants varies from study to study and few studies separate short-term migrants from longer-term migrants. Therefore, a migrant was defined as, *“An individual who is residing in a foreign country, irrespective of the causes, voluntary, involuntary, and the means, regular or irregular, used to migrate*” [12].

### Data sources and search strategy

A protocol for the conduct of the review of reviews was developed a priori*.* Using the same search strategy two authors (CC, KP) separately searched MEDLINE, Web of Science, PubMed, PsycINFO and the Cochrane Library for Systematic Reviews on the mental health of first generation migrants that were published in English. Searches were conducted in November 2015 without applying date restrictions.

Firstly, a broad (free text) string search “Migrant$” OR “Immigrant$” AND “Systematic review” was applied to the databases. This broad search was supplemented with a detailed search (see Additional file 1). The detailed search did not uncover any further relevant articles beyond those reviews that were retrieved using the initial broad-based search. Therefore, the outcome of the broad search will be reported in this review of reviews. Reference lists of systematic reviews that were deemed eligible for inclusion were searched for any reviews which were not retrieved in the database searches. Primary authors were asked to provide any missing key information when required. Two reviewers (CC, KP) used the eligibility criteria independently to screen titles and then abstracts and, finally, the papers that passed title and abstract screening were then obtained in full-text form.

### Inclusion criteria

The following criteria were used to determine the suitability of systematic reviews for inclusion in this review of reviews:

1. Types of studies: Systematic reviews with a quantitative assessment of the mental health or wellbeing of first generation migrants or a quantitative assessment of factors explaining differences in the mental health or wellbeing of first generation migrants.
2. Types of participants: Systematic reviews of male and female first generation migrants were considered for inclusion as well as children and adults. Reviews which included mixed generations were included only if data on first generation migrants could be extracted and analysed separately from other generations.
3. Types of mental health disorders: All mental health disorders (such as depression, anxiety and psychosis) along with general mental health, symptoms, distress and wellbeing.
4. Types of measures: psychiatric diagnoses (DSM V or ICD 8,9,10) and validated measures such as the Beck Depression Inventory (BDI) and General Health Questionnaire (GHQ).
5. Quality Scale: Systematic reviews with and without a quality scale.
6. Language: Systematic reviews published in English.

### Exclusion criteria

1. Systematic reviews that focused on co-morbidity with substance abuse, learning disability or neurological disorders.
2. Mixed methods systematic reviews from which quantitative data on migrant mental health could not be extracted and analysed separately from qualitative data. Systematic reviews of migrant mental health which included mostly qualitative studies were excluded as these reviews would be unable to quantify the risk/prevalence of mental-ill health in first generation migrants and the associated risk factors.
3. Systematic reviews which focused on the physical health of first generation migrants with a mental health condition and did not add any new insights about mental health.
4. Systematic reviews which focused on migrants in detention, prison or psychiatric facilities.

### Study quality assessment

Review quality was rated independently by CC and KP using AMSTAR (A Measurement Tool to Assess Systematic Reviews). AMSTAR has good inter-rater reliability and content validity and assesses reviews on 11 categories including publication bias, quality assessment and methods of combining results [13]. AMSTAR total scores were used to weight the findings of the review (0–4: low quality; 5–8: moderate quality; and 9–11: high quality).

### Data extraction

Data were extracted from included reviews into a pre-designed form by CC. Fields included details about samples and sampling, migration course, countries, assessment measures and status, quality appraisal outcome, results and conclusion. A randomly selected 20 % of data extraction forms were checked and reviewed independently by a second review (KP). The concordance with these between both reviewers was 100 %, however if this had not been the case a third reviewer would have been invited to review the 20 % of data extraction forms and discussions between these three reviewers would have taken place until consensus was reached.

### Analysis

The Preferred Reporting Items for Systematic Reviews and Meta-analysis (PRISMA) guidelines [14] directed the conduct of this review. The reviews did not have sufficient methodological quality to conduct a quantitative summary of results. Instead, the reviews were synthesised in narrative form. The results were analysed according to the reason for migration and type of mental health disorder.

## Results

Searches identified 1820 (1819 via databases and 1 via citation searching) potentially relevant articles and 1787 after the removal of duplicates. The review of 1787 titles and then 71 abstracts yielded eight papers that met the inclusion criteria for full review (Additional file 2).

### Characteristics of included reviews

The characteristics of the included reviews are summarised in Additional file 3. A full list of the excluded studies with reasons are presented in Additional file 4. Overall, the included reviews comprise 74,251 first generation migrants (adult and children), including refugees, asylum seekers and labour migrants. Most reviews focused on migrants who migrated to high income countries, most frequently the United Kingdom, Canada, Australia, Sweden and the USA. Several reviews used meta-analytic analyses to pool prevalence rates of mental health in first generation migrants and to assess the association between risk factors and the likelihood of mental illness.

### Quality assessment of included studies

Of the eight reviews, three reviews attained a moderate AMSTAR score (range 5–8) and five reviews were rated to be low quality (scoring ≤4) (see Table 1).

**Table 1:** Quality rating of review using AMSTAR

Study	1. Priori design provided	2. Duplicate study selection & data extraction	3. Comprehensive literature search	4. Status of publication an inclusion criteria	5. List of studies provided (Included &Excluded)	6. Characteristics of studies provided	7. Scientific quality of studies assessed	8. Scientific quality used appropriately in formulating conclusions	9. Appropriate methods used to combine study findings	10. Likelihood of publication bias assessed	11. Conflict of interest included	Overall Quality rating
Borque et al. [6]	✓	✓	✓	×	×	✓	✓	✓	✓	✓	×	8/11 (moderate)
Das Munshi et al. [16]	✓	×	✓	×	×	✓	✓	×	✓	✓	×	6/11 (moderate)
Nilaweera et al. [19]	✓	×	✓	×	×	✓	✓	×	×	×	×	4/11 (low)
Cantor Graee & Selten [8]	✓	×	×	×	×	✓	×	×	✓	×	×	3/11 (low)
Lindert et al. [9]	✓	×	×	×	×	✓	×	×	✓	×	×	3/11 (low)
Fazel et al. [17]	×	×	×	×	×	✓	×	×	✓	×	×	2/11 (low)
Bronstein and Montgomery [15]	✓	×	✓	✓	×	✓	✓	✓	×	×	×	6/11 (moderate)
Slewa-Youghan et al. [18]	×	✓	✓	×	×	✓	✓	×	×	×	×	4/11 (low)

Of the three moderate quality reviews, one reported the prevalence of CMDs specifically in children and young people [15] another reviewed the risk of psychotic disorders [6] and the third review appraised the impact of social mobility on the development of CMDs in first and second generation migrants [16]. These three reviews had a priori design procedures, comprehensive search strategies, provided study characteristics and assessed scientific quality of the studies. The AMSTAR items that these three reviews ‘failed’ to provide were a full list of included and excluded studies as well as not declaring whether or not there was any conflict of interest.

Of the five low quality reviews, one reported the prevalence of CMDs and psychotic disorders of adult and children first generation migrants [17], one reported the prevalence of CMDs [9], another reported the risk of schizophrenia [8], one reported on PTSD and depression in Iraqi refugees [18] and the fifth reported the prevalence of postpartum depression (PPD) [19]. All five reviews presented the characteristics of included studies. AMSTAR scores were poor because reviews failed to report how duplicate study selection was addressed as well as omitting details about: the status of publication as an inclusion criterion, a list of included and excluded studies, if there had been any assessment of publication bias and if there was any conflict of interest. In addition, only one review used a method of assessing quality [19].

### Common mental disorders for first generation migrants in general

Two reviews reported on the prevalence of CMDs in first generation migrants in general. Lindert et al. [9] found high prevalence rates of depression (35 %), anxiety (28 %) and PTSD (47 %) among first generation migrants (n = 24,051). Most rates were based on self-report questionnaires and scales such as the Hopkins Symptom Checklist and the Harvard Trauma Questionnaire.

Nilaweera et al. [19] reported on PPD. This mixed methods review included ten quantitative studies and reported clinical (diagnostic) outcomes such as ICD 9 as recorded in medical notes and self-report outcomes such as the Edinburgh Postnatal Depression Scale (EPNDS). The studies which used the EPNDS for the most part reported similar prevalence rates of PPD between 11.2 and 19.1 %. Only one study in this review specifically calculated prevalence rates of PPD using ICD-9 codes recorded in medical notes and this study reported very low prevalence rates of PPD at just 5 %. Seven of the ten quantitative studies in this review by Nilaweera et al. [19] reported specifically on PPD in South Asian born women and prevalence rates varied considerably from 3 to 52 %, with a mean prevalence of 19 %. In this review by Nilaweera et al. [19] three other quantitative studies reported on the prevalence of PPD in women born overseas in general and prevalence rates ranged from 5 to 19 %, with a mean prevalence of 12 %.

### Common mental disorders in asylum seekers and refugees

Fazel et al. [17] reported clinical outcomes that were approximately similar to the general population: 5 % (99 % CI 4–6 %) for major depression and 4 % (99%CI 3–6 %) for anxiety. The PTSD for refugees was 9 % (99 % CI 9–10 %) for adults and 11 % (99 % CI 7–17 %) for children.

Most studies (21/35) in the review by Lindert et al. [9] focused on refugees who had a reported prevalence of 44 % (95 % CI 27–62 %) for depression and 40 % (95 % CI 17–64) for anxiety as assessed mainly by self-report instruments commonly the Hopkins-Symptom-Checklist (HSCL-25) and the Harvard-Trauma-Questionnaire (HTQ). Collectively, 19/35 studies on refugees in the review by Lindert et al. [9] found a prevalence for PTSD of 36 % (95 % CI 23–49 %).

Bronstein and Montgomery [15] found that, on average, 18 % of refugee children experienced self-reported depression (range: 3–30 %) and 36 % had PTSD (range: 9–54 %). Findings from the review by Slewa-Younan et al. [18] indicate that, on average, 43 % of Iraqi adult refugees experienced self-reported depression (range: 28–75 %) and 25 % had PTSD (range: 8 to 37 %).

### Psychotic disorders in first generation migrants in general

The relative risk of clinically diagnosed psychotic disorders in first generation migrants compared to the settled population was 2.3 (95 % CI 2.0–2.7) according to Borque et al. [6] and 2.7 (95 % CI 2.3–3.2) in the review by Cantor-Graee and Selten [8].

### Psychotic disorders in asylum seekers and refugees

The review by Fazel et al. [15] was the only review that provided prevalence data for psychotic disorders among asylum seekers and refugees − 2 % (99 % CI 1–6 %).

#### Risk factors for mental ill-health

Three of the eight reviews provided a quantitative assessment of risk factors for mental ill-health in first generation migrants [6, 9, 16]. These reviews assessed economic factors (GNP of the host country and downward social mobility), socio-demographic factors (gender) and geographical factors (host country, urbanisation, country of origin) as possible contributors to mental ill-health in first generation migrants.

### Country of origin

According to Borque et al. [6] first generation migrants from countries where the majority of the population were categorised as ‘Black’ had a higher risk for psychotic disorders (IRR 4.0 95 % CI 3.4–4.6) than migrants from countries where the majority of the population were categorised as ‘White’ (IRR 1.8, 95 % 1.6–3.1) or ‘other’ (IRR 2.0 95 % 1.6–2.5). The countries included in this categorisation were not specified.

### Host country

Borque et al. [6] found that first generation migrants to the UK (IRR 2.8 95 % CI 2.2–3.5), the Netherlands (IRR 2.5 95 % CI 2.0–3.2) and Scandinavia (IRR 2.3 95 % CI 1.9–2.7) had a similar risk of experiencing a psychotic disorder. Migrants to Israel were less likely (IRR 1.5 95 % CI 1.1–2.1) to experience a psychotic disorder than migrants to these countries/regions.

### Gross National Product (GNP)

Lindert et al. [9] reported an inverse relationship between GNP of a host country and lower prevalence of depression among labour migrants (14 % in higher GNP host countries vs. 31 % in lower GNP host countries). However, the prevalence of depression among refugees was similar irrespective of host country GNP (40 % in higher GNP host country compared to 42 % in lower GNP host country).

### Social mobility

A moderate quality review by Das Munshi et al. [16] conducted a random effects meta-analysis of the association between downward social mobility and CMDs in first generation migrants. Although the review included studies on first and second generation migrants, the meta-analysis only included studies on first generation migrants and findings suggested that first generation migrants who experienced downward social mobility were more likely to screen positive for CMDs than those migrants who maintained a stable socio-economic position or those who were upwardly mobile (Crude OR: 1.56; 95 % CI 1.04–2.33). There was evidence to suggest that there was a greater association between downward social mobility and CMDs in refugees or asylum seekers (Crude OR: 1.98, 95 % CI 1.06–3.73) than in labour migrants (Crude OR: 1.15 95 % CI 0.87–1.50).

### Gender

Borque et al. [6] found no difference in the incidence rate ratios for first generation male and female migrants for psychotic disorders (*males* IRR 2.1, 95 % CI 1.7–2.6; *females* IRR 2.4, 95 % CI 1.9–2.9).

### Urbanisation

According to findings by Borque et al. [6] the study setting, urban or mixed urban rural did not impact on the IRR for psychotic disorders (*mixed urban/ rural* IRR 2.2, 95 % 1.9–2.6; *urban* 2.7, 95 % CI 2.0–3.6).

## Discussion

The present review of reviews is to our knowledge the first to systematically identify and synthesise a wide range of evidence on the prevalence or risk of mental ill-health among first generation migrants, as well as identifying a range of potential risk factors to explain differences in mental ill-health between first generation migrants and the settled majority. The findings indicate higher levels of mental illness in first generation migrants, but the lack of high quality reviews affects the inferential capacity of these findings, and as such suggests the need for better, more robust methodologies when conducting research and reviews on the mental health of first generation migrants.

### Common mental disorders (CMDs) in first generation migrants

Five reviews provided data on the prevalence of CMDs (depression, anxiety, PTSD, PPD) in first generation migrant and all reported a significantly higher level of one or more CMDs in one of the migrant populations included. These findings indicate that first generation migrants’ need for mental health care may be greater than that of the settled majority population. Although a small proportion of the global population, these findings are of great relevance given disproportionate suffering and lack of accessible and culturally appropriate mental health care [20]. In addition, recent international socio-political crises indicate that the need to escape war, poverty and lack of opportunity will continue. This likely increased need for mental health services combined with migrants underutilisation of mental health services and delay in accessing help is a cause for concern [21].

The prevalence of PPD was only reported in one review, where it was reported to be 19 % in South Asian women, and 12 % in women born overseas in general. However, prevalence rates did vary considerably, ranging from 3 to 52 % across included studies. This large variation in prevalence rates may be explained by the fact that several different screening questionnaires for PPD were used in included studies and none of these questionnaires have been validated for detecting PPD in South Asian migrant women. Therefore, caution is advised around these findings. Another factor which could have impacted on the reported prevalence rates of PPD in South Asian migrant women is the fact that the screening questionnaires (eg. EPNDS) used were translated into different languages with no evidence of the testing of the comprehensibility of the translated version with South Asian migrant women. Recently, a systematic review was conducted into the reliability and validity of the EPNDS for detecting perinatal common mental disorders (eg. PPD) among women in low-and lower-middle-income countries which included South Asian women. This review reported that just one of 16 included studies that used a translated version of the EPNDS met all criteria for culturally sensitivity, meaning the comprehensibility of the translated versions of the questionnaire in the remaining 15 studies was unclear [22]. Therefore, ensuring the use of cultural sensitive and validated screening questionnaires for assessing migrant mental health in research studies needs prioritised. Without the use of validated culturally sensitive outcome measures it is difficult to make any conclusions regarding migrants’ risk of mental ill-health.

Despite the issues identified in the review by Nilaweera et al. [19] the available evidence indicates that the prevalence of PPD is particularly elevated in South Asian female migrants compared to prevalence rates reported in general population studies which indicate a prevalence rate of PPD of approximately 11 % [23]. However, even though it seems likely that certain female migrants are at increased risk of PPD, to date there has been little attempt by researchers to quantitatively asses risk factors which may explain this increase. Findings from the few individual studies which have been conducted indicate that lack of social support, language barriers and differences in how female migrants recover after birth and care for their infants may in part explain elevated risks of PPD [24–26]. The findings of the present review suggest that health professionals should endeavour to ensure appropriate screening for PPD for female migrants particularly South Asian migrant women and implement strategies to reduce the likelihood of this debilitating condition developing, given the potential for the significant negative consequences of PPD for both mother and baby [27, 28].

The prevalence of PTSD reported by Lindert et al. [9] for first generation migrants in general (47 %) was significantly elevated compared to the prevalence of PTSD in the settled majority population. Data acquired from surveys indicates that 1–2 % of the general population from various countries across the globe experience PTSD at some point, although this has inevitably been found to be higher in conflict affected countries such as in Northern Ireland, at 8.8 % [29, 30]. Similarly, our findings indicate that refugees and asylum seeker adults have a higher prevalence of PTSD, both Slewa-Younan et al. [18] and Lindert et al. [9] reported elevated levels of PTSD in refugees, 25 % and 36 % respectively. Prevalence rates for PTSD for refugee and asylum seeker children (36 %) were also significantly higher than prevalence rates from general adolescent populations which have been reported to be around 5 %. No specific population studies for younger children were identified [31]. While most of the reviews presenting prevalence data on PTSD in refugees/asylum seekers [9, 15, 18] indicated significantly elevated prevalence rates of PTSD in this population compared to the general population, the review by Fazel et al. [17] reported only a moderate increase in PTSD rates.

Data on PTSD was only available from four systematic reviews each of which used different types of outcomes (clinical and non-clinical); therefore, it is difficult to determine exactly what the risk of PTSD is among first generation migrants. Fazel et al. [17] was the only review which included studies with PTSD confirmed solely with a diagnostic interview. Therefore, given that a diagnostic interview is likely to be the most thorough and valid method in determining the presence of PTSD, it would be reasonable to suggest that the lower estimates of risk of PTSD (9 % of refugees) may be more accurate, compared to higher estimates (36 %) as reported by Lindert et al. [17], who reported mainly on self-report measures. More generally, research indicates that self-report of mental disorders can lead to overestimates of the prevelances compared with diagnostic interviews and clinical diagnoses [32, 33]. Furthermore, there is a general consensus that self-report measures are more likely to be subject to bias. For example the patient can answer questions according to how they feel they should respond [34]. Contrary to this diagnostic interviews are conducted by trained clinicians in mental health, psychology, or psychiatry and therefore it is much more difficult for patients’ difficulties to be misrepresented.

Nevertheless, it should be noted that PTSD may present differently in different cultures, and that such a diagnostic methodology may not capture the extent of post-traumatic reactions in this population. However, even if the lower prevalence of PTSD (9 %) as reported by Fazel et al. [17] is to be considered more accurate it still indicates an increase in risk for PTSD among first generation migrants. This is not surprising given that pre-migration experiences as well as the experience of migration, particularly for those fleeing war, are often characterised by severe and prolonged exposure to violence, abuse and terror.

Lindert et al. [9] reported significantly higher levels of depression in first generation migrants in general (35 %) and for refugee/ asylum seeker adults (44 %) compared to estimates of prevalence in the general population reported to be between 8 and 12 % [31, 35]. Slewa-Younan et al. [18] reported similarly high levels of depression (43 %) to Lindert et al. [9] in Iraqi refugees. Prevalence rates for child refugee/asylum seeker as reported by [15] were also elevated (18 %) when compared to the general population of children (3 %) and adolescents (5.6 %) [36], although it is important to bear in mind that for many of the reviews reporting high rates of depression in migrant populations, self-reported and non-validated measures were used. However, in one review [17] prevalence rates for depression in adult refugees/asylum seekers were reportedly lower than in the general population. These conflicting findings make it difficult to draw any firm conclusions on the prevalence of depression in first generation migrants, although for the most part the available evidence suggests that it is higher. Possible explanations for lower prevalence rates reported by Fazel et al. [17] may be related to the fact that this review only included clinical diagnostic measures of mental disorders, for which the accuracy of such measures is difficult to ensure especially in non-western refugees for whom the validity of psychiatric measures developed in western populations might be restricted. For example terms frequently used in diagnostic instruments in the USA such as “feeling down” or “blue” when translated directly to another language may yield meaningless expressions [37]. Furthermore, Fazel et al.’s [17] lower prevalence rates of depression in refugees may be explained by the strict criteria for depression which clinical diagnostic measures often have, therefore it is possible that these measures may only pick up on higher levels of depression and exclude non-clinical low mood which self-report measures may have captured. A further plausible explanation for the lower prevalence of depression reported in the review by Fazel et al. [17] may be related to fear of stigma by study participants, most of whom were from Vietnam. In many Asian cultures mental illness is highly stigmatised and those who suffer mental illnesses are often discriminated against, and are thought to bring shame on their family [38]. These cultural differences certainly could have affected participant’s responses in studies and lead to under-reporting of symptoms.

There was also conflicting evidence on whether prevalence rates for anxiety were higher or lower in first generation migrants compared to the settled majority population. Fazel et al. [17] report a 5 % prevalence of anxiety in refugees/asylum seekers, much lower than the prevalence reported by Lindert et al. [9] of 40 % in refugees and asylum seekers and 28 % in migrants in general. The prevalence rates reported by Fazel et al. [17] were very similar to estimates of prevalence from the settled majority, estimated to be around 4.7 % [39]. Differences in the country of origin on migrants in the reviews by Fazel et al [17] and Lindert et al. [9] could explain the wide variation in anxiety prevalence rates reported by these authors. For example, Lindert et al. [9] included migrants from several African countries in which there has been extreme poverty and conflict such as Somalia and Sierra Leone, whereas the review by Fazel et. Al [17] did not include any African countries. Therefore, it is conceivable that refugees from countries where there have been huge socio-political issues may be more likely to experience anxiety. Additionally, anxiety and for that matter depression may be legacy issues related to reasons for migration and may also be because of factors related to the host country such as poor social support [40]. Like the findings for the prevalence of depression, it is not entirely clear whether prevalence of anxiety is higher or lower in first generation migrants, but for the most part the evidence would suggest an increased risk of anxiety in first generation migrants. This difference in prevalence rates may also be explained by the respective definitions of anxiety. Fazel et al. [17] included studies that reported solely on Generalised Anxiety Disorder (GAD) as defined by the DSM, whilst Lindert et al. [9] reported on anxiety in general, including the use of questionnaires based on either the DSM or the International Classification of Diseases (ICD). This distinction is likely to have influenced the prevalence rates, as the DSM-IV and -V criteria for GAD is fairly precise (3 specific symptoms for more than 6 months), such that those suffering from GAD symptoms that do not meet the diagnostic threshold, those suffering from other forms of anxiety disorder (Panic Disorder, Agoraphobia, Specific Phobias, Social Anxiety, Obsessive Compulsive Disorder), or those suffering from a similar disorder to GAD that is understood differently in the country of origin, would not be included. Additionally, Fazel et al. [17] included only refugees, and as mentioned mostly from Vietnam. This is in stark contrast to Lindert et al. [9] who included refugees and asylum seekers, from a wide range of countries experiencing pervasive and brutal armed conflicts and socio-economic emergencies such as Somalia, Sudan, and Iraq. Arguably, the inclusion of asylum seekers, who are under great psychological strain facing the fear of deportation, and individuals migrating from countries with ongoing conflict, makes up a substantially different population. In this context, it is unsurprising that Fazel et al. [17] found a lower prevalence rate. Moreover, cultural differences in how mental illness is viewed in different countries as discussed previously in relation to depression may have played a role in the lower prevalence rates of anxiety reported by Fazel et al. [17].

### Psychotic disorders in first generation migrants

The risk of psychotic disorders in first generation migrants in general was reported in two reviews [6, 8] to be two to three times higher compared to the settled majority a finding supported by an emergent body of evidence suggesting migration may be a risk factor for psychotic disorders [41]. However, Fazel et al. [17] reported relatively low prevalence rates for psychotic disorders in refugees at 2 %, similar to the estimates of the lifetime prevalence of psychotic disorders reported in the general population (3 %) [42]. Nevertheless, it is important to draw attention to the fact that that the estimate of prevalence of psychotic disorders in refugees by Fazel et al. [17] was only based on two studies of 226 people, whereas the estimates of risk of psychotic disorders in first generation migrants were based on approximately ten times as many studies and participants. Furthermore, one of the studies [43] noted that although only 2.3 % of the sample of Vietnamese refugees in Norway, suffered from a psychotic disorder at the time of interview, 5 % had suffered a ‘psychotic break’ at some point since migration, almost 15 years previously (p.365). However, it may be that refugees suffer from a smaller increase in risk of psychotic illness than other disorders, namely depression and PTSD. This may be explained by the environmental risk factors associated with the differential disorders. The aetiology of depression is heavily influenced by experiences of loss, interpersonal trauma and life stress [44] and PTSD is by definition influenced by direct exposure to, or witnessing, a traumatic event [45]. These risks are intertwined with the experiences of those seeking asylum. Psychotic illness however, has a much stronger genetic heritability, and has environmental risk factors that include heavy cannabis use and pervasive experience of childhood abuse or neglect [46]. These experiences are much less likely to be higher in asylum seeking populations. However, an increased risk of psycotic disorders in migrants in general is still likely, given the risk factors of isolation and exposure to discrimination in developing psychotic illness [20].

The prevalence of psychotic disorders in first generation migrants is therefore unclear, and is likely to be affected by a number of wider issues. Firstly, increased rates of psychotic disorders found in first generation migrants may be affected by racist stereotyping in which clinicians diagnose psychotic disorders more frequently in ethnic minority populations than the majority population. This is a longstanding theory in psychiatry as one of the possible causes for the higher prevalence of psychotic disorders found in ethnic minority populations, and some research studies have concluded that ethnic minorities may be given a psychotic disorder diagnosis even though they are suffering from a non-psychotic mood disorder [47, 48]. This is explained by clinician’s failure to recognise differences in symptom expression, misinterpreted patient wariness, stereotyping and cultural insensitivity [47, 49]. However, other research has highlighted higher prevalence of psychotic disorders in minority populations even without these effects. Research that blinds clinicians to ethnic backgrounds has maintained the finding of higher prevalence rates [50], and recent research has linked this to higher rates of disadvantage, poverty and lack of opportunity in ethnic minority groups [51] as well as lack of social support, isolation, exposure to discrimination and lack of access to culturally appropriate services [52].

Given the present conflicting findings it would make sense to consider those estimates of risk which reported on larger sample sizes to be more accurate [6, 8]. The high risk estimates of psychotic disorders in the larger studies indicate an increase in psychotic disorders among migrants which signifies the need for health professionals to consider migration experiences as possible risk factors for psychotic disorders. The importance of this is further amplified when considered in the context of other well-established risk factors such as childhood trauma, cannabis use, maternal complications and urbanicity [53] which would seemingly present a similar risk magnitude as to those found for migration in this review of reviews.

### Factors which may influence the risk of mental ill-health in first generation migrants

African and Caribbean first generation migrants were found to be at much greater risk of psychotic disorders. Supporting this finding, a meta-analysis by Torteilli et al. [41] reported that black African and Caribbean migrants had an almost five times greater risk of suffering from a psychotic disorder than the white British majority. This increased risk has been extensively researched and may be explained by institutional racism in mental health service provision, disproportionate levels of poverty and social exclusion, reduced social capital and high exposure to prejudice and discrimination [52] as well as the ethnic density effect in which individuals have a greater risk of mental ill health if they live in a neighbourhood in which they are in a minority ethnic group. The protective element of the ethnic density effect is thought to offer a buffering effect through strong social support networks, [16] a reduction in the frequency of exposure to racism and access to culturally and religiously appropriate services [52].

Our review findings indicated that downward social mobility is associated with an increased risk of CMDs in first generation migrants. Downward social mobility is reportedly common among newly arrived migrants as it can be difficult for migrants to resume the occupation they had on their home country and some are forced to take jobs of lower status than their country of origin [54]. Our findings are supported by other research: for example, a study of over 3000 migrants to the USA also found evidence that downward social mobility increases the risk of a mental health problem in migrants [55]. This study reported that a loss of at least three steps in subjective social status of migrants was associated with an increased risk of a depressive episode among Latino and Asian migrants to the USA. Possible explanations for this may include a decline in personal autonomy, control and self-respect, as well as possible self-blaming for the occupational decline, all of which may lead to stress, depression and substance use [54].

Host country GNP, which relates to the possibility that a country could provide paid jobs, only impacted on the depression and anxiety rates of labour migrants and not on refugees. This is unsurprising given differences in motivation for migration; reasons for migration of labour migrants searching paid employment greatly differ from reasons of migration of refugees and asylum seekers who are by definition searching a place of safety.

An interesting finding from our review of reviews was the difference in the risk of psychotic disorders in migrants to Israel compared to migrants to the UK, Netherlands and Scandinavian countries. A possible explanation for this is the differences in immigration history in these countries. The introduction of the Law of Return in 1950 granted every Jewish person the automatic right to immigrate to Israel and become a citizen of the state. This has brought continuous migration to Israel to the present day with 31 % of those residing in Israel being migrants [56]. Therefore, the lower risk of psychotic disorders in migrants to Israel compared to migrants to the UK, Netherlands and Scandinavia could be explained by the fact that migrants to Israel are most likely people with Caucasian Jewish ancestry, who have moved from minority to majority status, and may be less likely to be considered ‘outsiders’ and discriminated against as a consequence [52]. This is supported by studies that found that Jewish Black African migrants have worse mental health than other migrant groups than the settled Jewish majority in Israel [44]. Furthermore, there is mounting evidence to suggest an association between perceived discrimination and incidence of psychotic disorders in ethnic minority groups [57–59]. For example, Veling et al. [47] found that the incidence of schizophrenic disorders in ethnic minority groups exposed to high levels of discrimination was much higher than in ethnic minorities exposed to very low levels. Equally, Becares et al. [58] found that adjustment for exposure to racism explained to a great extent the higher incidence of schizophrenia in low ethnic density neighbourhoods.

In addition to the findings on factors that influenced the mental health of first generation migrants, it was surprising to find that urban living did not appear to have an impact on the rates of psychotic disorders in first generation migrants. Previous research has indicated that urban living was a risk factor for schizophrenia in specific migrant groups, as well as being a well-established risk factor for psychotic disorders in the general population [8, 60]. This discrepancy may be explained by the poor range of urbanicity in the methodology, with no ‘rural only’ categorisation, reducing statistical sensitivity. This has been reported as a common limitation in previous studies [61].

The risk factors (eg. downward social mobility) identified for mental ill-health among migrants would be worthy of qualitative research in the future. This may be helpful for gaining a better understanding of the risk factors and may help in the development of both policy and clinical interventions to tackle these risk factors.

### Strengths and limitations of this review of reviews

The major strength of this review is that it is the first to synthesis the evidence on the risk/prevalence of mental health and associated risk factors for first generation migrants. Therefore, the present findings provide a better understanding for migrants’ needs for mental health care, as well as factors which may impact on this need. Furthermore, the present review of reviews draws attention to the inadequate quality of existing systematic reviews on migrant mental health and the lack of inclusion of risk factors affecting migrant mental health, issues which need to be addressed without delay.

Limitations of this review include the sub optimal quality of included reviews with no high quality reviews identified, as well as the lack of breakdown of prevalence/risk of mental ill-health and associated risk factors according to generation and reason for migration. Systematic reviews that failed to distinguish between generations had to be excluded resulting in a reduction in the overall number of reviews that could be included. The grouping of first generation migrants as one large arbitrary group in many of the systematic reviews seriously restricts our ability to make any conclusions about the mental health of first generation migrants. Separate analysis of prevalence/risk of mental ill-health associated risk factors by generation are likely to be more meaningful given the known differences in experiences between the migrant generations. Research has shown that pre-migration experiences, the reason for migration, the migration journey and post-migration experiences, such as residency status and acculturation, can all impact upon the mental health of migrants [7]. In contrast, second generation migrants will not have been affected directly by the actual migration process, but may experience different stressors related to identity, prejudice and discrimination [4].

Another major limitation was the fact that most included reviews did not separate first generation migrants by country of origin. Mixing migrants together without separation or consideration of the country of origin is a serious limitation given the socio-economic, historical, political and other (e.g. language) differences between countries. Additionally, the lack of quantitative assessment of the factors associated with differences in first generation migrants’ mental health seriously restricted the ability to make any convincing conclusions about risk factors. Finally, the exclusion of non-English systematic reviews means that the findings of several systematic reviews were not included or represented in this review of reviews.

The use of screening questionnaires which were not validated for the population under investigation within included reviews further limits the validity of the findings. Culturally sensitive validated screening questionnaires are essential to allow comparisons of contexts and to allow identification of risk and protective factors for mental health problems. On one hand, using diagnostic interviews based on the DSM or ICD provide highly structured evidence-based diagnoses, but on the other the evidence base is derived mostly from Western countries with specific social constructions of mental illness strongly rooted in the medical model [62]. Such a model may not be relevant to the definition of mental disorders globally. Equally, self-report screening tools may be more reflective of individual meaning and symptomology but they are less structured and this leaves more room for bias, socially desirable answers and differing interpretations. Even if validated within the target population and thus culturally and linguistically relevant, there remain difficulties in comparing results cross culturally. For example, the widely used Impact of Events Scale (IES) and the Child Impact of Events Scale (CIES) have been translated into numerous languages e.g. [63] and their psychometric properties have been researched in just as many countries and minority populations [64, 65]. However, despite this, the IES/CIES is at best a measure of self-reported symptoms of trauma and not a proxy for a PTSD diagnosis. This is even more relevant given changes to the definition of PTSD since the 5th Edition of DSM was published in 2013, including the removal of criterion A2 - fear, helplessness and horror - which has been shown not to improve diagnostic accuracy [45]. This review of reviews has highlighted some wide estimates of prevalence rates, largely due to differences in the method of assessment. Lower prevalence rates tended to be reported for studies using structured clinician’s assessment, and this is likely to reflect conceptual differences between psychiatric disorders and self-reported psychosocial distress. Such a distinction is important in future cross-cultural research on mental health, and clearer definitions with matched assessment methods will help to provide quantifiable data for comparison across cultures, languages and countries.

Finally, the inclusion of only quantitative studies could be considered a limitation of this review of reviews, as there are qualitative systematic reviews which also provide important insights into migrant mental health. For example Sullivan and Rehm [66] conducted a systematic review on the mental health of undocumented migrants using mainly qualitative studies which identified themes which could explain why undocumented migrants could be very vulnerable to mental illness. These themes included limited resources; marginalization/isolation and vulnerability/exploitability. However, despite the important insights that qualitative systematic reviews could provide regarding migrant mental health, our review of reviews aimed to quantify migrants’ risk of mental ill-health and the associated risk factors, and qualitative reviews would be unable to acheive this.

### Implications for practice and policy

Notwithstanding the limitations detailed above, the current review of reviews provides policy makers and health professionals with a stronger evidence base for the disproportionate burden of mental ill-health of first generation migrants. The findings can help inform policy makers when making decisions about mental health treatment resources and service accessibility, health professionals in identifying and treating individuals at risk of mental ill-health and third sector organisations and migrants themselves in creating awareness of disproportionate risk, normalising psychological distress and improving help-seeking behaviour.

The findings also suggest that a greater emphasis is required on providing preventative psychosocial interventions to first generation migrants, particularly as there is evidence of the effectiveness of such interventions in reducing the risk of developing mental ill-health in refugees and asylum seekers [67]. The availability of these preventative interventions could considerably reduce the future need to access more intensive and costly secondary care mental health interventions, not to mention the human cost of suffering from severe and preventable mental health disorders [68].

Despite our findings indicating a likely higher prevalence of CMDs and psychotic disorders in first generation migrants, it is disconcerting to note that research suggests that migrants underutilise mental health treatment [21]. Policy makers and service managers may need to examine primary care services and develop strategies to reduce barriers for migrant groups in accessing such services as well as improving awareness of these services in the first place. The details of this review of reviews’ findings should provide some insight into how and where to target these improvements in service access and provision.

### Directions for future research

This review has identified several future areas for research:

1. The need for the conduct of higher quality studies and reviews in the area
2. The need for further quantitative research to assess factors associated with differences in first generation migrants’ mental health
3. The use of culturally sensitive validated instruments to measure the mental health of migrants
4. Separation of first generation migrants from other migrants when conducting research studies on migrant mental health
5. Separation of first generation migrants by country of origin in research studies
6. The need for qualitative research into the risk factors for migrant mental ill-health

## Conclusion

In conclusion, the findings of this review of reviews suggest that first generation migrants including refugees/asylum seekers are at increased risk of mental ill-health, including CMDs and psychotic disorders. However, as the body of evidence is of sub-optimal quality and there were some inconsistencies in findings between reviews, caution around this finding is advised. Inconsistencies in prevalence rates may be explained by different approaches to assessment (self-report and diagnostic interview) as well as the use of screening questionnaires which had not been validated in the population under investigation. Downward social mobility, host country, immigration to a host country with a low GNP and country of origin were all associated with an increased risk of mental ill health in first generation migrants. Health professionals working with migrant communities should consider migration as a possible risk factor for mental ill health whilst taking into consideration that migrant communities are not a homogenous group, and that some migrant groups may be at greater risk of mental ill health than others. It is the migrant groups at the greatest risk of mental ill health that most attention and resources should be directed towards to prevent and reduce inequalities in mental health.

## Additional files

Additional file 1: Broad Search Strategy. Contains details of the broad search strategy used to complement a more narrow search strategy reported upon in this paper. (DOCX 13 kb) Additional file 2: Flow diagram of review stages. Contains details of the various stages of the review. (DOCX 265 kb) Additional file 3: Characteristics of included studies. Contains important details about included reviews characteristics including the country of origin, outcome measures used and findings. (DOCX 32 kb) Additional file 4: List of excluded studies. Contains the titles and authors of reviews which were excluded along with reasons for exclusion. (DOCX 20 kb)

## Abbreviations

AMSTAR: A measurement tool to assess systematic reviews
CI: Confidence interval
CIES: Child impact of events scale
CMD: Common mental disorders
DSM: Diagnostic and statistical manual
EPNDS: Edinburgh Postnatal Depression Scale
GAD: General anxiety disorder
GNP: Gross National Product
HSCL: Hopkins-Symptom checklist
HTQ: Harvard Trauma Questionnaire
ICD: International Classification of Diseases
IES: Impact of events scale
IRR: Incidence risk ratio
PPD: Postpartum depression
PTSD: Post-traumatic stress disorder
