# Supplementary material for: The mental health and wellbeing of first generation migrants: a systematic-narrative review of reviews
Source: Global Health. 2016 Aug 25;12(1):47. doi: 10.1186/s12992-016-0187-3 (PMC4997738; doi:10.1186/s12992-016-0187-3)
Supplement: Additional file 1: — Broad Search Strategy. Contains details of the broad search strategy used to complement a more narrow search strategy reported upon in this paper. (DOCX 13 kb) [file 12992_2016_187_MOESM1_ESM.docx]

Additional file 1: Detailed search strategy total hits (n= 20, 837)

1. Review
2. “Migrants” OR “immigrants”
3. “ethnic$”
4. “Mental”
5. “Psych$”
6. “Wellbeing” or “health
7. 1 AND 2 OR 3 AND 4 OR 5 OR 6
