# Supplementary material for: The mental health and wellbeing of first generation migrants: a systematic-narrative review of reviews
Source: Global Health. 2016 Aug 25;12(1):47. doi: 10.1186/s12992-016-0187-3 (PMC4997738; doi:10.1186/s12992-016-0187-3)
Supplement: Additional file 2: — Flow diagram of review stages. Contains details of the various stages of the review. (DOCX 265 kb) [file 12992_2016_187_MOESM2_ESM.docx]

Additional file 2: Flow diagram of review stages
