# Supplementary material for: The mental health and wellbeing of first generation migrants: a systematic-narrative review of reviews
Source: Global Health. 2016 Aug 25;12(1):47. doi: 10.1186/s12992-016-0187-3 (PMC4997738; doi:10.1186/s12992-016-0187-3)
Supplement: Additional file 3: — Characteristics of included studies. Contains important details about included reviews characteristics including the country of origin, outcome measures used and findings. (DOCX 32 kb) [file 12992_2016_187_MOESM3_ESM.docx]

Additional file 3: Characteristics of included systematic reviews and meta-analyses

***Common mental health disorders***

| Author | Study type | Migration details | Countries/continents | Outcome measures | Mediators | Quality assessment | Results | Conclusion |
| --- | --- | --- | --- | --- | --- | --- | --- | --- |
| Nilaweera et al.2014 | Systematic review | Women who had migrated from South Asian countries to high income countries  Age of women not specified. | Migrating from:  Pakistan  India  Bangladesh  Sri lanka  South Asia-country not specified  Migrating to:  Canada  Australia  UK  Norway  USA | Postpartum depression (PPD) screening scale,  Diagnostic interview with a nurse psychotherapist,  Modified CES-D,  EPNDS,  Research diagnostic criteria for depressive illness Goldberg’s standardised psychiatric interview,  DSM V-cut of >10,  Physician diagnosis-recorded ICD-9 diagnosis codes,  Scale for clinical assessment in clinical neuropsychiatry | No quantitative analysis on interaction factors and mental health disorders among first generation migrants, but a narrative overview of interaction factors in relation to increased PPD in migrant women was provided. | Mirza and Jenkins point checklist assessing if the study had a clear aim, adequate sample size, clear inclusion/ exclusion criteria, valid measurement of mental health, reported on response rate and losses, adequate description of data and statistical methods. Average score was 7.5/9 | 15 studies included-10 quantitative and 5 qualitative.  Quantitative studies total sample size was 102,427 of which 25,183 were immigrant women including 5427 South Asian immigrants. 5 /10 quantitative studies reported a two fold increase in PPD (OR 1.8-2.5) and 7/10 provided prevalence data on PPD for South Asian women which indicated that between 2.9-52% of South Asian migrants suffered PPD, with a mean prevalence of 19% . Three studies did not provide subgroup analysis and presented the prevalence of PPD in women born of overseas in general who were found to have a prevalence of PPD which ranged from 5% to 15% with a mean prevalence of 12%. | Migrant women from South Asia to high income countries have a high prevalence of PPD. |

**Abbreviations:** DSM- Diagnostic and Statistics Manual. ICD, International Classification of Diseases, EPNDS- Edinburgh Postnatal Depression Scale, PPD-Postpartum Depression

| Author | Study type | Migration details | Countries/continents | Outcome measures | Mediators | Quality assessment | Results | Conclusion |
| --- | --- | --- | --- | --- | --- | --- | --- | --- |
| Das Munshi et al 2012 | Systematic review and meta-analysis | 1^st^ and 2^nd^ generation migrants including labour migrants and asylum seekers adults and children | Migrating from: Scandinavia, Finland, Turkey, Former Soviet Union, Europe, Middle East, North America, Iran, Iraq, South East Asia, Central/ South America, Puerto Rico, outside Scandinavia/ Europe  Migrants to: USA, Canada, NZ, Sweden, Netherlands, Israel | -HSCL  -CIDI  -10 item depression inventory  -Dutch self-report questionnaire for internalising/ externalising behaviour  -12-item Chinese health questionnaire  -20 item scale for somatic and psychological items  -9 item self-concept scale  13 item alienation sale  -SCL-90 (revised)(2g)  -Lagner scale  -GHQ 12  -PERI-D  -CES-D  -Sengal health scales  -SRQ  -DIS  -Vietnamese depression scale | Social mobility and common mental health disorders | A point checklist assessing  -study type, response rate, sampling method, sample size, attrition rates, methods of defining migrant groups and assessment of confounders and interactions  I^2^ statistics to assess heterogeneity <25% low, 50% (moderate) 75% (high) | 12 studies included, 10 focusing on first generation and 2 focusing on second generation representing a migrant population of 18, 854 of which 5179 were included in a random effects meta-analysis. Migrants (1^st^ generation migrants only) with downward social mobility were more likely to screen positive than migrants who maintained their upwardly socially mobile or maintained their position.  An odds ratio (OR) of 1.56 (95% CI-1.04-2.33) for common mental health disorders for refugees and asylum seekers (combined) experiencing downward social mobility.  For refugees/ asylum seekers the OR was 1.15 (95% CI 0.87-1.50), and for labour migrants the OR was 1.98 (95% CI 1.06-2.37) | Downward social mobility appears to be associated with an increased risk of common mental health disorders in first generation migrants. The association is greater for labour migrants compared to refugees /asylum seekers |

**Abbreviations:** HSCL-Hopkin Symptom Checklist, CIDI-Composite International Diagnostic Interview, SCL-symptom checklist, GHQ-General Health Questionnaire, PERI-D Psychiatric Epidemiology Research Interview Demoralization Scale, CEDS-Centre for Epidemiological Studies Depression Scale, SRQ-Self Report Questionnaire, DIS-Diagnostic Interview Schedule ;Odds Ration-OR

| Author | Study type | Migration details | Countries/ continents | Outcome measures | Mediators | Quality assessment | Results | Conclusion |
| --- | --- | --- | --- | --- | --- | --- | --- | --- |
| Fazel et al. 2005 | Systematic review | Refugees resettled in Western countries both adults and children | Migrating from:  South East Asia  Former Yugoslavia  Central America  Middle East  Migrating to:  Australia  Canada  Italy  Norway  New Zealand  UK  USA | The diagnostic interview schedule  the structured clinical interview for the diagnostic and statistical manual, the composite international diagnostic interview,  the present state examination,  the post-traumatic symptom scale, the clinician-administered post-traumatic stress disorder scale, the schedule for affective disorders and schizophrenia, the schedule for affective disorders and schizophrenia for school-age children, diagnostic interview schedule for children, the anxiety disorders interview schedule-revised | Not assessed quantitatively | None reported | 20 eligible surveys provided results for 6743 adult refugees  In the larger studies, 9% (99% CI 8–10%) were diagnosed with post-traumatic stress disorder and 5% (4–6%) with major depression, with evidence of psychiatric comorbidity. Five surveys  of 260 refugee children yielded a prevalence of 11% (7–17%) for post-traumatic stress disorder. | Refugees resettled in Western countries could be about ten times more likely to have post-traumatic stress disorder than the age-matched non-refugee population, whereas they were less likely to have depression. |

| Author | Study type | Migration details | Countries/ continents | Outcome measures | Mediators | Quality assessment | Results | Conclusion |
| --- | --- | --- | --- | --- | --- | --- | --- | --- |
| Lindert et al.2009 | Systematic review & meta-analysis | Refugees and first generation  Labour migrants(adults mixed gender)  Age range 16-88 | Migrating from:  Latin America-e.g. Puerto Rico, Somalia, Burna, Italy, Ethiopia, Sierra Leone, Afghanistan, Iran, Sudan, Iran, Iraq, Korea, Cambodia, Bosnia, Guatemala, Vietnam, China, Kosovo, Butan, multiple countries in the Caribbean  Migrating to:  USA, Thailand, UK, France, Argentina, Canada, Gambia, Netherlands, Iran, Uganda, Australia, Netherlands, China, Croatia, Australia, Sweden, Nepal | WMH survey  CIDI  HSCL25  HTQ  Beck depression  CIDIS  HOS  Personal resource questionnaire  Self- perception scale  PAI  CEDS  GHQ28  DIS  PDS  GAF  AUDIT  War trauma questionnaire  Anxiety inventory | Economic factors (Gross National Product-GNP) and depression in migrants | None-though a reason for not doing this is provided-“No appropriate assessment scale available” | 35 studies included-most of which focused on refugees (n=20). Review represents 24,051 migrants.  For all migrants grouped together depression rates were 27%, anxiety rates were 19% and PTSD prevalence was20%.  For refugees depression prevalence rates were 44, anxiety rates were 40%and PTSD rates were 36%. For labour migrants prevalence rates were 20% and anxiety rates were 21%  Differences in depression prevalence rates were found according to host country GNP. For example labour migrants who migrated to a host country with a GNP greater than 30,000 had depression rates of 14% compared to those labour migrants who migrated to a lower GNP country where depression was 31%. This was not found for refugees. | Prevalence rates of Common Mental Health Disorders are high in first generation migrants and host country GNP may play a role in this. |

Abbreviations: WMHS-World Mental Health Survey, CIDI- Composite International Diagnostic Interview, HSCL-Hopkins Symptom Checklist, CEDS-Centre for Epidemiological Studies Depression Scale, GHQ-General Health Questionnaire, PAI-Personality Assessment Inventory, PDS- Posttraumatic Stress Diagnostic Scale, GAT-Global Assessment of functioning, AUDIT-Alcohol Use Disorders Identification Test

| Author | Study type | Migration details | Countries/ continents | Outcome measures | Mediators | Quality assessment | Results | Conclusion |
| --- | --- | --- | --- | --- | --- | --- | --- | --- |
| Bronstein and Montgomery 2011 | Systematic review | Refugee children and young people under 25 migrating to a Western country | Migrating to:  Canada, Demark,  Netherlands,  Sweden  UK  USA  Migrating from:  58.4% of migrant children where from African countries e.g.: Somalia, this was followed by 20.7% from the Middle Eastern countries including the Persian Gulf. 11.4% were from Europe 6.6% were from Asia, .0.5% were from South American and 2.4% were unidentified. | HTQ  IES  PTSD checklist  RATS  UCLA PTSD index  HSCL 37A  Strengths and difficulties questionnaire  YSR  GHQ 28  CHQ  Traumatic symptom checklist for children | None assessed quantitatively | Reporting quality was judged by applying the guidelines set by the STROBE initiative. STROBE criteria is used to ensure research is high quality and in this review only those studies which met the STROBE criteria were included | In total 22 studies were retrieved, however for 8 of these the sample was contained in another study, therefore 14 studies of 3003 children/ young people were included.  Seven studies with 2124 children/young people reported on PTSD and reported a prevalence range for PTSD of 19-54%, with a mean prevalence of 36%. Three studies of 599 children/ young people reported on the prevalence of depression, and found that the prevalence of depression ranged from 3%-30% with a mean prevalence of depression of 18%. Other studies in the review reported on internalising and externalising behaviour which were not deemed relevant for analysis for this review of reviews. | Refugee children/ young people appear to experience high levels of psychological stress, such as depression and PTSD. |

Abbreviation: HTQ- Harvard Trauma Questionnaire, IES-Impact of Events Scale, PTSD=Post-Traumatic Stress Disorder, RATS-Reaction of Adolescents to Traumatic Stress, UCLA-University of California and Los Angeles, HSCL-Hopkins Symptom Checklist, YSR-Youth Self Report, GHQ-General Health Questionnaire, CHQ-Child Health Questionnaire.

| Author | Study type | Migration details | Countries/ continents | Outcome measures | Mediators | Quality assessment | Results | Conclusion |
| --- | --- | --- | --- | --- | --- | --- | --- | --- |
| Slewa-Younan et al.2014 | Systematic review | Refugee adults resettled in a Western country | Migrating to:  Netherlands,  Sweden  USA  Austrialia  Migrating from:  Iraq | HSCL-25,  PTSD checklist-Military version,  Clinician administered PTSD scale for DSM-IV,  Primary Care PTSD screen,  Primary Care Evaluation of Mental disorders  WHO CIDI  Clinician administered PTSD scale | None assessed quantitatively | Methodological quality was assessed using an adapted criteria from Reijnder et al.2008 | Prevalence of PTSD and depression among Iraqi refugees resettled in Western countries was higher than prevalence rates determined from community samples. For PTSD prevalence rates ranged from8-37.2 %( mean 25%) and depression prevalence rates ranged from 28.3%-75 %( mean 43%). | Iraqi refugees resettled in Western countries have elevated levels of PTSD and depression compared to the non-Iraqi refugee population. |

Abbreviation: PTSD=Post-Traumatic Stress Disorder,; HSCL-Hopkins Symptoms Checklist, WHO-World health organisation, CIDI- composite International Diagnostic Interview; DSM- Diagnostic and Statistics Manual

***Psychotic disorders***

| Author | Study type | Migration details | Countries/ continents | Outcome measures | Mediators | Quality assessment | Results | Conclusion |
| --- | --- | --- | --- | --- | --- | --- | --- | --- |
| Cantor and Graee and Selton 2005 | Systematic review & Meta-analysis | First generation migrants and second generation (SGM) migrants analysed separately- includes a sample of mainly first generation migrants  . | Migrating to:  UK  Israel  Denmark  Canada  Netherlands  Sweden  Australia  Migrating from:  Caribbean  Africa  Asia  Suriname  Netherlands  Turkey  Finland  Scandinavia  Middle East  Greenland  UK  Former Soviet Union | Diagnosis of schizophrneia, paranioa, schizophnreiaffective disorder,  !CD 9. Research Diagnostic criteria, CATEGO Schizoprehnia, paranioa or other, DSM IV, ICD 8, ICD 10 | No specific quantitative analysis on interaction factors and mental health disorders among first generation migrants.  However the review did conduct analysis on interaction factors for first generation migrants and SGMs combined. | The internal  validity of each study was assessed based on methodological  features and potential for selection bias, information bias and confounding. Each study attained  a numeric quality score, using the scale from a prior systematic review of schizophrenia incidence studies. They conducted a broad  quality appraisal and classified studies into higher, average and lower quality ranges. | A meta-analysis of 61 effect sizes for 2156 first generation migrants yielded mean-weighted incidence rate ratios (IRRs) of 2.3 [95% confidence interval (CI) 2.0–2.7] for first generation migrants. There was no significant risk difference between generations, but there were significant differences according to ethnicity and host country for first and second generation migrants grouped together.  5 studies were rated high quality and 4 of these 5 related to first generation migrant. | First generation migrants are at increased risk of psychotic disorders compared to their native counterparts. Ethnicity and host country were mediators of psychotic disorders, but there was no between first and other generations of migrants and future researchers may wish is distinguish between these groups. |

**Abbreviations**- SGM-Second Generation Migrants, DSM- Diagnostic and Statistics Manual. ICD, International Classification of Diseases,

| Author | Study type | Migration details | Countries/ continents | Outcome measures | Mediators | Quality assessment | Results | Conclusion |
| --- | --- | --- | --- | --- | --- | --- | --- | --- |
| Borque et al.2011 | Systematic review | 1^st^ and 2^nd^ generation male and female migrants (second generation separated for analysis from first generation). No age restriction or reason for migration specified. | Migrating to:  UK  Sweden  Israel  Denmark  Australia  Canada  North America  Migrating from:  Caribbean  Africa  Asia  Europe  Former Soviet Union  North America  South America  Australia  Middle East  Unknown | DSM-IV, non-affective and  affective psychoses  ICD-10, schizophrenia ICD-9 and -10, schizophrenia Average  ICD-8 and -10, schizophrenia  DSM-IV, schizophrenia and  related disorders  DSM-IV, schizophrenic disorders  ICD-9 and -10, schizophrenia and  other psychoses  DSM-IV, psychotic disorders  ICD-8 and -10, schizophrenia  DSM-IV, schizophrenia and other  non-affective psychoses  ICD-9, schizophrenia  RDC, schizophrenia  Unspecified, schizophrenia and paranoia  Unspecified, schizophrenia  ICD, schizophrenia  Schneiderian diagnosis criteria for schizophrenia | Gender, country of origin, category/ minority status, urbanization setting | The internal  validity of each study was assessed based on methodological  features and potential for selection bias, information bias and confounding. Each study attained  a numeric quality score, using the scale from a prior  systematic review of schizophrenia incidence studies  6 low quality, 10 average quality and 5 high quality. | 21 studies met study inclusion criteria, representing 5508 (first generation migrants ) and 4422 (SGM) cases. A meta-analysis of 61 effect sizes for first generation migrants of 2.3 [95% confidence interval (CI) 2.0–2.7] for first generation migrants. There were significant differences according to ethnic group and host country. The  mean-weighted IRR for first generation migrants from areas where most of  the population were black was 4.0 (95% CI 3.4–4.6) versus  1.8 (95% CI 1.6–2.1) for groups classified as ‘white’  and 2.0 (95% CI 1.6–2.5) for groups classified as ‘other’. No difference in psychotic disorders was found by gender *males* IRR 2.1, 95% CI 1.7-2.6; *females* IRR 2.4, 95% CI 1.9-2.9) or by urbanisation, (*mixed urban/ rural* IRR 2.2, 95% 1.9-2.6; *urban* 2.7, 95% CI 2.0-3.6). | First generation migrants are at increased risk schizophrenia and related disorders. In particular there was a very high incidence of schizophrenia and related disorders among black migrants (first generation migrants).  Gender and urbanization setting did not increase the risk of psychotic disorders in first generation migrants |

**Abbreviations** SGM-Second Generation Migrants, DSM - Diagnostic and Statistics Manual. ICD, International Classification of Diseases, RDC-Research Diagnostic
