# Supplementary material for: The mental health and wellbeing of first generation migrants: a systematic-narrative review of reviews
Source: Global Health. 2016 Aug 25;12(1):47. doi: 10.1186/s12992-016-0187-3 (PMC4997738; doi:10.1186/s12992-016-0187-3)
Supplement: Additional file 4: — List of excluded studies. Contains the titles and authors of reviews which were excluded along with reasons for exclusion. (DOCX 20 kb) [file 12992_2016_187_MOESM4_ESM.docx]

| **Additional file 4: Excluded studies**  **Author** | **Year** | **Reason for excluding** |
| --- | --- | --- |
| Abebe et al. | 2014 | mixed generation - data by generation not presented or able to be analysed separately. |
| Agorastos et al. | 2012 | not a SR |
| Alemi et al. | 2014 | no quantitative data |
| Anikeeva et al. | 2010 | focus on health not mental health |
| Aspinall | 2002 | not a SR |
| Bak-Klimek et al. | 2015 | not focusing on first generation |
| Beister et al. | 1995 | not a SR |
| Belhadj et al. | 2014 | mixed generation - data by generation not presented or able to be analysed separately. |
| Borque et al. | 2012 | not a SR |
| Bourque, Francois; Malla, A. | 2009 | not a full paper conference abstract |
| Clare CA(1), Yeh J. | 2012 | not a SR |
| Collins et al | 2011 | not a SR |
| Cooper | 2005 | not a SR |
| Crafa D(1), Warfa N. | 2015 | second generation with MH problems |
| Dealberto | 2013 | not a SR |
| Fung K(1), Dennis CL. | 2010 | not a SR |
| Gagnon AJ(1) et al. | 2009 | focus on health not mental health |
| Ginieniewicz and Mc Kensie | 2014 | unclear about generation |
| Guarnaccia PJ(1), Lopez S. | 1998 | not a SR |
| Hansen E, Donohoe M. | 2003 | not a SR |
| Hansson et al. | 2012 | no English full text |
| Jaeger et al. | 2012 | Mixed generation - data by generation not presented or able to be analysed separately. |
| Kalt et al. | 2013 | focus on health not mental health |
| Kirmayer LJ et al. | 2011 | focus on health not mental health |
| Koch | 1988 | focus on health not mental health |
| Koch E(1), Müller MJ. | 2008 | not a SR |
| Lassetter and Callister | 2009 | focus on health not mental health |
| Lee KH(1), Woo H. | 2013 | not a SR |
| Lindert et. Al | 2008 | not a SR |
| Lindert et. Al | 2011 | not a full paper conference abstract |
| Lindert et. Al | 2008 | no English full text |
| Linscott and Van Os | 2013 | unclear about generation |
| Bursztein et al.2010 | 2010 | not a SR-no presentation of included studies |
| Maffla | 2008 | focus on health not mental health |
| McCauley | 2005 | not a SR |
| Mirsky et al | 2008 | not a SR |
| Peeters RF. | 1985 | focus on health not mental health |
| Porru et al. | 2014 | not a SR |
| Porter and Haslam | 2005 | not focusing specifically on migrants includes internally displaced |
| Pottie K(1), Dahal G, Georgiades K, Premji K, Hassan G. | 2014 | focus not on MH |
| Reed et al. | 2012 | not focusing specifically on migrants includes internally displaced and refugee's |
| Robjant et al. | 2009 | focus on detainees |
| Seeman MV(1). | 2011 | not a SR |
| Selkirk M, Quayle E, Rothwell N. | 2014 | focus on help seeking |
| Siwarhara et al. | 2014 | not focusing specifically on migrants includes internally displaced |
| Steel et al. | 2009 | not focusing specifically on migrants includes internally displaced |
| Stevens GW(1), Vollebergh WA. | 2008 | mixed generation |
| Sullivan and Rehn | 2005 | focus on qualitative outcomes |
| Tahira A(1), Agius M. | 2012 | not a SR |
| Tarricone et al. | 2012 | focus on ethnicity not migrants |
| Torteilli et al | 2015 | mixed generation - data by generation not presented or able to be analysed separately. |
| Uscher Pines | 2008 | not focusing specifically on migrants/ focus on health |
| Van Os et al. | 2009 | unclear about generation |
| Veling | 2013 | not a SR |
| Veling and Susser | 2011 | not a SR |
| Yearwood EL(1), Crawford S, Kelly M, Moreno N. | 2007 | not a SR |
| Bhugra et al | 2003 | not a SR |
| Hamlyn,J.; Duhig,M.; McGrath,J.; Scott,J. | 2013 | not a SR |
| Janas | 2004 | focus on health not mental health |
| Neale and Wand | 2013 | not a SR |
| Dealberto | 2010 | not a SR |
| Mc Grath | 2005 | mixed generation - data by generation not presented or able to be analysed separately. |
| Saha et al. | 2004 | mixed generation - data by generation not presented or able to be analysed separately. |

Abbreviations: MH=Mental health, SR=Systematic review
